# Association of ABO/Rh Blood Groups and ABO Gene Variants *rs8176746* and *rs495828* with COVID-19 Severity Among Egyptian Patients

**DOI:** 10.3390/v18070805

**Published:** 2026-07-22

**Authors:** Marwa H. Elnagdy, Alshimaa Magdy, Ahmed Hazem El-Nagdy, Eman Ali Elkordy, Ahmed E. Taha, Atef Elmougy, Mohamed Ayed Rashwan, Basil Mohammed Alomair, Anas Adi, Waleed Eldars, Mohamed Elgamal, Mohamed Ahmed Noureldin, Mayada Sabry Zeid, Ali Sobh

**Affiliations:** 1 Department of Medical Biochemistry and Molecular Biology, Faculty of Medicine, Mansoura University, Mansoura City 35516, Egypt; marwaelnagdy85@mans.edu.eg (M.H.E.); alshimaamagdy82@yahoo.com (A.M.); 2 Department of Basic Medical Sciences, Faculty of Medicine, New Mansoura University, New Mansoura City 35516, Egypt; wellydars@gmail.com; 3 Department of Microbiology, Faculty of Dentistry, Horus University, Damietta El Gadeeda City 34517, Egypt; ahazem@horus.edu.eg; 4 Department of Anatomy and Physiology, College of Medicine, Imam Mohammad Ibn Saud Islamic University (IMSIU), Riyadh City 11461, Saudi Arabia; 5 Microbiology and Immunology Unit, Department of Pathology, College of Medicine, Jouf University, Sakaka City 72388, Saudi Arabia; 6 Department of Medical Microbiology and Immunology, Faculty of Medicine, Mansoura University, Mansoura City 35516, Egypt; 7 Department of Paediatrics, Mansoura University Children’s Hospital, Faculty of Medicine, Mansoura University, Mansoura City 35516, Egypt; atef2075@gmail.com (A.E.); dr_mayadasabry@mans.edu.eg (M.S.Z.); ali.sobh@mans.edu.eg (A.S.); 8 Department of Clinical Pathology, Faculty of Medicine, Mansoura University, Mansoura City 35516, Egypt; dr_mohammed1982@mans.edu.eg; 9 Department of Internal Medicine, College of Medicine, Jouf University, Sakaka City 72388, Saudi Arabia; bmalomair@ju.edu.sa; 10 Medical Officer, Dar Al Shifa Hospital, Hawally City 13034, Kuwait; 11 Department of Chest Medicine, Mansoura University Hospitals, Faculty of Medicine, Mansoura University, Mansoura City 35516, Egypt; melgamal6@gmail.com; 12 Department of Paediatrics, Faculty of Medicine, Horus University, Damietta El Gadeeda City 34517, Egypt; mnoureldin@horus.edu.eg

**Keywords:** ABO, COVID-19, pathogenesis, *rs495828*, *rs8176746*, SARS-CoV-2, SNPs, viruses

## Abstract

The link between ABO/Rh blood groups and COVID-19 severity remains a topic of debate. We aimed to investigate the role ABO/Rh blood groups, *rs8176746* and *rs495828* single nucleotide polymorphisms (SNPs), in COVID-19 severity among COVID-19 patients in Mansoura City, Egypt. A total of 306 Egyptian patients were included in this cross-sectional study. They were grouped into 86 cases with non-severe infection and 220 with severe infection according to clinical, laboratory, and radiological findings. ABO/Rh blood groups were confirmed. A TaqMan real-time PCR SNP genotyping assay was used to examine the allelic discrimination of *rs8176746* and *rs495828* SNPs. Older age, male gender, and the presence of comorbidities were associated with severe COVID-19. AB blood group was significantly associated with a protective effect against severe COVID-19 (*p* = 0.029) in the unadjusted analysis. There was no statistically significant association between Rh type and disease severity. GT genotype was significantly associated with a higher frequency of AB blood groups in both *rs8176746* and *rs495828* (*p* < 0.001). However, there was no statistically significant association between the studied SNPs and COVID-19 severity. After adjusting for age, gender, and the presence of comorbidities, the protective effect of the AB blood group with severe COVID-19 did not remain significant. No association was observed between Rh type *rs8176746* and *rs495828* SNPs and COVID-19 severity. The impact of ABO/Rh blood types and related SNPs on COVID-19 severity can vary by the ethnic group and population being studied. This diversity emphasizes the need for specialized research approaches to better understand the demographic, clinical, and genetic factors that lead to variations in COVID-19 outcomes among various groups.

## 1. Introduction

The most catastrophic pandemic of the past ten years is thought to be the coronavirus disease 2019 (COVID-19) [1]. The WHO reports that, as of 5 April 2026, there were 779,213,537 confirmed COVID-19 cases globally, including 7,114,282 deaths [2]. In Egypt, 516,023 confirmed COVID-19 cases, including 24,830 deaths, were reported by the WHO as of 5 April 2026 [3]. COVID-19 has variable presentations, ranging from mild or even no symptoms (mostly in children and young adults) to severe disease with respiratory failure that requires mechanical ventilation (mostly in elderly people with underlying comorbidities) [4]. Severe acute respiratory syndrome coronavirus-2 (SARS-CoV-2), the causative organism of COVID-19, has a spike (S) protein that consists of two subunits, S1 and S2. The S1 protein expresses a receptor-binding domain that binds to the angiotensin-converting enzyme 2 (ACE2) receptor found in human cells. Meanwhile, with the S2 glycoprotein, the virus can fuse and invade the cell [5].

The ABO blood group system, discovered over 100 years ago, is the main system used in blood transfusion. Based on the agglutination patterns of their red blood cells (RBCs), people are divided into four major groups: A, B, O, and AB. The A and B antigens on red blood cells and the associated antibodies in the sera of people who do not have those antigens make up the ABO blood group system. RBCs, as well as the surfaces of other tissues and secretions, have ABO antigens. Immunocompetent people begin to naturally create anti-A and anti-B immunoglobulins around six months of age [6]. A robust association between ABO antigens and different systemic diseases has been recorded for infectious diseases, cardiovascular diseases, and cancer [7], and is a well-established determinant of thrombotic disease risk [8].

The link between the ABO/Rh blood group versus SARS-CoV-2 infection and COVID-19 severity remains a topic of debate and is not fully understood. Accordingly, it is a hot spot for global research [9,10,11,12,13,14,15,16,17,18,19,20,21,22,23,24,25,26,27,28,29,30,31,32,33,34,35,36,37,38,39,40,41,42,43,44,45,46,47,48]. Several studies reported important associations between the ABO/Rh blood group versus SARS-CoV-2 susceptibility/disease severity. On the other hand, some international research reported lack of significant associations in this regard, as presented in Appendix A, Table A1.

The receptor-binding domain within the spike protein was found to significantly mimic human galectin, especially glycan A [49]. Thus, it was hypothesized that people with O or B groups who have anti-A antibodies will be at less risk of infection than patients with group A or AB. This was attributed to the neutralizing effect of anti-A antibodies against infection [50]. Moreover, soon after the pandemic, Zhao and colleagues first documented that non-O blood group carriers are at increased risk of COVID-19 in comparison to O blood types [51].

The *q34.2* region of chromosome 9 contains the blood group ABO gene, which codes for a glycosyltransferase that creates the unique A or B blood group antigen by adding the last monosaccharide to a glycoconjugate, resulting in glycan antigen formation with naturally occurring polyclonal antibodies to these defined antigens [6]. Certain single-nucleotide polymorphisms (SNPs) of this gene have been linked in recent research to cardiovascular disorders [52], hypertension [53], and SARS-CoV-2 infection/severity [54,55,56,57,58,59,60,61,62,63]. Moreover, genome-wide association studies (GWAS) in Chinese populations found that *rs8176746* and *rs495828* polymorphisms in the ABO gene are strongly related to ACE activity [64]. Therefore, it was hypothesized that these polymorphisms may have an important effect on COVID-19 severity, as the virus depends mainly on the ACE2 receptor for host–cell invasion.

Studying the relationship between blood groups and COVID-19 severity is challenging, since severity and prognosis rely on numerous factors, including the presence of comorbidities, disease stage, and immune system status [65]. Thus, it is necessary to clarify the link between ABO/Rh blood groups and COVID-19 severity in various populations. In this context, the current study aimed to investigate the role of ABO/Rh blood groups, *rs8176746* and *rs495828* SNPs, on COVID-19 severity among COVID-19-confirmed Egyptian patients at Mansoura City, Egypt.

## 2. Materials and Methods

### 2.1. Design of the Study

The Institutional Research Board of the Faculty of Medicine at Mansoura University in Egypt approved the study protocol (RP.20.05.70) on 10 May 2020. This research adheres to the ethical standards of the 1975 Declaration of Helsinki. We obtained written informed consent from all participants. For adults, this consent was obtained from the patient themselves or from a legally authorized representative. For children, consent was obtained from the child’s parents/legal guardians, with assents where appropriate. The sample size was calculated using an online (Raosoft) sample size calculator (https://raosoftcalculator.com/, last accessed on 1 March 2022) with a response distribution of 50%, confidence level 95%, margin of error of 5.61%, and a population of 607,500 in Mansoura City, Dakahlia Governorate, Egypt, during 2021 [66].

**Figure 1:**
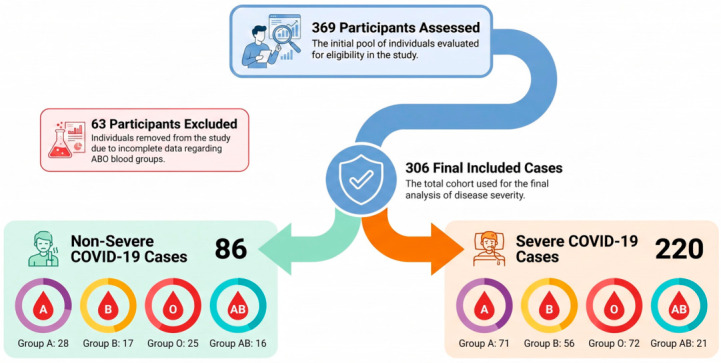
Flow diagram of participant inclusion.

This cross-sectional study was carried out between August 2020 and March 2022. Detailed medical history, including age and gender, type of blood group (A, B, O, AB), and clinical examination, were performed for all subjects under study to detect comorbidities, signs of infection, and complications. Participants without complete ABO blood group information or those with unconfirmed COVID-19 diagnosis were excluded from the study. The study included 306 Egyptian COVID-19 patients that were confirmed by real-time reverse-transcription PCR (rRT-PCR) in at least one biological sample (Figure 1). Non-severe COVID-19 cases totaled 86, and severe COVID-19 cases totaled 220 patients. COVID-19 severity was defined according to the WHO’s Clinical Management of COVID-19: Interim Guidance [67]. All included cases were not vaccinated. Patients were excluded if they had any active viral infections like HBV, HCV, and HIV, autoimmune diseases, or previous history of documented SARS-CoV-2 infection. During the study period, all severe COVID-19 cases admitted to the Mansoura University Hospital Isolation Unit, Mansoura City, Egypt, were included according to their clinical, radiological, and laboratory data, including complete blood count, coagulation marker (D-dimers), and inflammation-related parameters (ESR, CRP, ferritin). The characteristics of non-severe versus severe patients are shown in Table 1. Non-severe cases were included from patients with symptoms that suggested non-severe illnesses like fever, sore throat, tiredness, cough, loss of taste, odor, muscle ache, bone ache, nausea, vomiting, diarrhea, chills, running nose, and conjunctivitis with a SARS-CoV-2 positive test [67].

### 2.2. Collection and Transport of Samples

Using aseptic techniques, blood samples were withdrawn cautiously from all patients on the 2nd day after the appearance of symptoms in mild cases, while in severe cases, samples were withdrawn on the 2nd day after admission and laboratory investigations were done. An amount of 5 mL of venous blood was withdrawn from all participants and then added in ethylenediaminetetraacetic acid (EDTA) tubes. The samples were transported in an ice bag for further processing at the Medical Biochemistry and Molecular Biology Department, Faculty of Medicine, Mansoura University. An amount of 2 mL was used for ABO/Rh blood testing, and the other 3 mL was used for genomic DNA extraction.

### 2.3. ABO/Rh Blood Testing

Blood grouping was confirmed using a direct testing method, as described before using blood group monoclonal anti-A, anti-B, anti-D antisera [68]. The absence of agglutination was confirmed by microscopic examination.

### 2.4. Genotyping of ABO rs8176746 (G/T) and rs495828 (G/T) SNPs

Following the manufacturer’s recommendations, genomic DNA was extracted from blood samples using a QIAamp DNA Extraction Mini Kit (Qiagen, Hilden, Germany). DNA purity and concentration were assessed using a NanoDrop 2000c spectrometer from Thermo Scientific (Waltham, MA, USA). DNA purity was accepted if the optical density at 260/280 was 1.7–2.0 and >1.5 at 260/230.

Genotyping of ABO *rs8176746* (G/T) and *rs495828* (G/T) SNPs was done using a TaqMan real-time PCR SNP genotyping assay (Azure Cielo 6, Azure, Dublin, CA, USA), as described by before [69]. The pre-designed TaqMan SNP Genotyping Assays that were used for allelic discrimination of the polymorphisms in ABO *Leu266Met rs8176746* and *ABO 5 UTR rs495828* were purchased from Thermo Fisher Scientific (assay IDs were C__25610772_30 and C__26744830_10, respectively). Each assay contained 2 different primers (forward and reverse) that flank the SNP site, as well as two TaqMan probes. Each one was labeled with either VIC or FAM fluorescent dyes that differ only at the site of the SNP. Regarding the *rs8176746*, the sequence was ACCGACCCCCCGAAGAACCCCCCCA[G/T]GTAGTAGAAATCGCCCTCGTCCTTG, while for *rs495828*, the sequence was CTGTAACTGTTGCAAGGGAGGTAAA[G/T]ATTTAGGTCATGAGTCCCTTCCATT. One probe complemented the wild-type allele, while the other complemented the mutant allele. For quality control, three water samples were used as blank controls in each run. Furthermore, to avoid genotyping errors, 10% of the samples were tested twice randomly, and every result was consistent.

### 2.5. Data Analysis

Collected data was revised, coded, tabulated, and entered into a computer using Statistical package for Social Science (IBM Corp., released 2017, IBM SPSS Statistics for Windows, Version 25.0., Armonk, NY, USA: IBM Corp.). The Hardy–Weinberg equation (HWE) equilibrium of the analyzed sample was investigated; *p*-value < 0.05 denotes non-consistency with HWE. The data was presented, and appropriate analyses were conducted based on the type of data for each parameter. The Kolmogorov–Smirnov test was used to assess normality; if the significance level was greater than 0.05, normality was assumed. Descriptive statistics were reported as mean ± standard deviation (SD) for numerical data and as frequency and percentage for non-numerical data. Student’s *t*-test was employed to evaluate the statistical significance of the difference between the means of the two study groups. The Chi-Square test (χ^2^) was utilized to examine the relationship between two or more qualitative variables. When more than 20% of the cells had an expected count of less than five, Fisher’s exact correction test (FET) was applied. The Mann–Whitney U test was employed to assess the statistical significance of the difference in a non-parametric variable between the two study groups. Univariate and multivariate logistic regression analyses were conducted to identify dependent and independent risk factors for a categorical outcome. In all the tests performed, *p*-values < 0.01 were classified as statistically highly significant, *p*-values < 0.05 as significant, and *p*-values ≥ 0.05 as non-significant.

## 3. Results

### 3.1. Participant Demographic and Clinical Data

The current study included 306 COVID-19 patients. Demographic and clinical data of the study participants are presented in Table 2. Their median age was 48 years, ranging from 2 months to 88 years. They were 38.2% males and 61.8% females. Hypertension and DM were the most prevalent comorbidities among COVID-19 patients, presenting at frequencies of 43.1% and 27.8%, respectively. The cases were categorized into non-severe (*n* = 86; 28.1%) and severe (*n* = 220; 71.9%) groups according to the clinical and radiological findings, following predefined WHO clinical criteria. Severe cases were significantly associated with decreased hemoglobin, leucocytosis, neutrophilia, lymphopenia, and platelet count, as well as increased CRP, SGOT, and creatinine, as shown in Table 3.

### 3.2. ABO/Rh Blood Testing

Regarding the frequencies of ABO/Rh blood groups among non-severe and severe COVID-19 cases, we found that the presence of AB blood groups was significantly linked with a protective effect against severe COVID-19 (*p* = 0.029), as listed in Table 4.

### 3.3. Genotyping of ABO rs8176746 (G/T) and rs495828 (G/T) SNPs

We studied two ABO gene polymorphisms: *rs8176746* and *rs495828*. HWE was used to examine potential participant variations related to the genotypes under study. As the *p*-value was greater than 0.05, there was no deviation between the observed and anticipated frequencies of the ABO *rs8176746* (*G*/*T*) and *rs495828* (*G*/*T*) genotypes. Table 5 shows the genotype frequencies in the studied cases. We found that the GG genotype represents 66.7% and 67.0% in *rs8176746* and *rs495828*, respectively. Table 6 analyzes each SNP according to ABO type. Regarding *rs8176746*, we detected that the *GG* genotype was significantly associated with a higher frequency of the A and O blood groups (96.0% and 95.9%, respectively) while the *GT* genotype was significantly associated with a higher frequency of the AB and B blood groups and *TT* with blood group B. Regarding *rs495828*, the GG genotype was significantly associated with a higher frequency of the B and O blood groups (95.9% and 89.7%, respectively), while *GT* genotypes were significantly associated with a higher frequency of the AB and A blood groups and the *TT* genotype with the A blood group. There was no statistically significant association between Rh with *rs8176746* and *rs495828* genotype, as shown in Table 7.

Genotypic and allelic distribution of *rs8176746* and *rs2495828* among the participants according to COVID-19 severity are presented in Table 8. There was no statistically significant association between both SNP and COVID-19 severity for both genotypes and alleles. Regression analysis for prediction of COVID-19 severity was presented in Table 9. In univariate analysis, older age, male gender, and the presence of comorbidities were identified as predictors of disease severity, while the AB blood group was protective in this unadjusted model. In multivariate analysis, only male gender and the presence of comorbidities remained as predictors of disease severity. Adjusted models for each SNP for age, gender, and comorbidities are presented in Table 10.

## 4. Discussion

There is debate and much complexity surrounding the relationship between ABO/Rh blood types versus susceptibility to SARS-CoV-2 infection and COVID-19 severity. The evidence is not yet conclusive, even though several studies point to possible links that might affect infection rates and disease consequences, as presented in Appendix A, Table A1. Consequently, more research in this field is still necessary to elucidate the underlying pathogenesis and public health implications. The conducted study aimed to investigate the impact of ABO/Rh blood groups, *rs8176746* and *rs495828* SNPs, on COVID-19 severity among Egyptian patients with COVID-19 in Mansoura City, Egypt. This is an observational, single-center, and exploratory study that found an unadjusted association of the AB blood group with a protective effect against the severity of COVID-19; we also found that male gender and comorbidities are robust predictors.

### 4.1. Participant Demographic and Clinical Data Versus COVID-19 Severity

In the conducted study, hypertension and DM were the most prevalent comorbidities among COVID-19 patients. Regression analysis, both univariate and multivariate, was used to predict COVID-19 severity. Severe COVID-19 was more prevalent among patients who were older, male, and had comorbidities. This agrees with the results of a study that determined the baseline characteristics and outcomes of patients infected with SARS-CoV-2 [70]. A study conducted in Saudi Arabia reported that exposure to SARS-CoV-2 infection is common among patients with type 2 diabetes, hypertension, cardiovascular, and chronic respiratory, and chronic inflammatory diseases, especially among males, and these patient groups require special care to prevent occurrence of COVID-19 complications [71]. According to a report from Egypt, young people without comorbidities exhibit a robust humoral immune response against SARS-CoV-2 [72]. There is limited knowledge about the immune responses to SARS-CoV-2 in children, who are typically asymptomatic or exhibit mild symptoms [73]. Cytokines and chemokines influence the child’s immunological response to SARS-CoV-2, affecting its initiation, duration, and downregulation. Even though COVID-19 is normally a minor condition in the pediatric population, certain children may experience severe clinical symptoms and require hospitalization, highlighting the need for further research [74]. In young people and those without cardiovascular conditions, blood group O clearly provided relative protection against SARS-CoV-2 susceptibility in Denmark [11]. In Pakistan, a strong relationship was noted between comorbidities (such as hypertension and diabetes) and severe outcomes in COVID-19 patients [47]. Research indicates that men may have larger levels of ACE2, a protein that allows viruses to infect human cells. ACE2 is present in multiple organs, including the lungs, heart, kidneys, blood vessel linings, and testes. This may explain why men are more prone to severe disease than women [75].

### 4.2. ABO Blood Group Versus COVID-19 Severity

SARS-CoV-2 outcomes may be attributed to the type of ABO blood group. We found that the AB blood group showed an unadjusted association with a protective effect against severe COVID-19 (*p* = 0.029) in older age male patients with associated comorbidities. These results are consistent with the results of researchers, who reported that blood type AB is linked to a favorable outcome. The research team reported that patients with A and B blood groups were more significantly impacted by severe COVID-19 compared to those with O and AB [75]. Similarly, in their meta-analysis, a research team, found that patients with blood group A are more vulnerable to COVID-19 infection and that blood type AB is linked to a lower risk of COVID-19 infection [76]. They observed that most complications were highly reported in group O, followed by A, and then B and AB. A study found that the lowest ratios of ICU admission (9.5%) and mortality (4.8%) were among the AB blood group [77].

In accordance with our results, after adjustment for age, gender, and the presence of comorbidities, a study from the USA reported that ABO blood type is not linked to the severity or mortality of COVID-19 [34]. Another study from the USA reported that ABO blood type was not linked to severe COVID-19 outcomes across the Delta, Alpha, and Omicron variants [38]. ABO and Rh blood types did not correlate with SARS-CoV-2 susceptibility or COVID-19 mortality in Saudi Arabia [37]. A recent Ethiopian study reported that the severity or mortality of COVID-19 is unrelated to variations in ABO blood types [42]. In Iran, ABO and Rh blood groups were not linked to the severity of COVID-19, ICU admission, or need for mechanical ventilation [46].

The pathogenesis underlying the association between different blood groups and COVID-19 severity might not be related to the ABO blood group itself. It could be linked to differences in ACE2 receptor expression, naturally occurring anti-A and anti-B antibodies, cytotoxic T cell response, and blood coagulation profiles. It was documented that, once SARS-CoV-2 infection occurs, infected individuals may produce antigens that are like ABO types that may induce anti-A and anti-B immunoglobulins [78]. Researchers suggested that if the virus spike proteins are produced in cells that already synthesize A and B antigens, the viral glycoprotein will carry the same A and B epitopes and would stimulate anti-B and anti-A production. These antibodies can prevent the binding of viral spike glycoprotein with cellular ACE2 receptors and the subsequent viral entry into the cell in a complement-dependent manner. They may also aid in the production of cytotoxic T cells. This leads to developing immunity to other viral antigens [79].

However, these mechanisms only support the protective role of blood group O [80], but cannot directly interpret the protection in AB individuals, who normally lack anti-A and anti-B antibodies, observed in our study. Indian research concluded that patients with blood group AB experienced severe COVID-19 [44,45]. Our study results do not support the above-mentioned reports.

Research confirmed the role of naturally occurring Anti-A antibodies in individuals with blood group O in protection against COVID-19 infection. In contrast, individuals with blood group A exhibit elevated ACE-1 activity, possibly resulting in more severe COVID-19 symptoms [65]. Therefore, the conflicting results in research may be multifactorial; they could arise from variations in populations and geographical locations, differences in the distribution of ABO groups across ethnicities, and the presence of factors such as comorbidities, age, or gender. Additionally, variants of SARS-CoV-2 may influence the findings of these studies.

### 4.3. Rh Blood Group Versus COVID-19 Severity

We found no statistically significant association between Rh blood groups and COVID-19 severity. Our results are in line with Turkish [26] and Indian [75] studies. On the other hand, it was reported that the risk of development of severe COVID-19 is decreased in Rh-negative blood group patients, suggesting that they are protected against severe SARS-CoV-2 infection [19]. Similarly, Rh-positive individuals were more susceptible to SARS-CoV-2 infection [21]. In Sudan, Rh-positive status has a positive effect on blood group O, but negatively impacts blood group A [25]. In Iraq, Rh-negative blood type provides a protective effect against SARS-CoV-2 [33]. It was reported that COVID-19 patients who had a negative Rh status were less likely to get infected, need to be intubated, or die [81]. However, there is no strong explanation as to why Rh-negative status is considered protective, particularly given that a higher percentage of the population is Rh-positive compared to Rh-negative individuals [82]. On the other hand, a study from the USA reported that Rh-negative patients have an increased risk of mortality [34].

### 4.4. The Studied SNPs Versus COVID-19 Severity

Host genetics can affect SARS-CoV-2 infection and COVID-19 severity. This may provide an important insight into the biological factors involved, paving the way to develop personalized treatment strategies for severe COVID-19 [83]. One potential pathogenesis that has been reported is the genetic polymorphism of the ABO blood system [65,83]. Thus, we aimed to evaluate the possible association between *rs8176746* G/T and *rs495828* G/T polymorphisms in ABO genes with COVID-19 severity in an Egyptian population. We found no statistically significant association between *rs8176746* or *rs495828* versus COVID-19 severity. Our results are in line with the results of Vargas-Alarcón and colleagues, who reported that there was no difference in the distribution of *rs8176746* and *rs495828* polymorphisms in Mexico [69]. In a Caucasian population, it was reported that *rs8176746* ABO SNP was not associated with the severity of COVID-19 [84]. Contrary to our results, Tanha research team reported that there was genome-wide association between *rs495828* in the ABO gene with severe COVID-19 risk [83]. ABO polymorphisms in *rs495828* and *rs8176746* lead to COVID-19 severity via increased ACE activity and cardiovascular disorders [50]. Moreover, it was documented that the GATC haplotype *rs8176746*–*rs8176740*–*rs495828*–*rs12683493* at the ABO locus is common in people with non-O blood groups and was shown to be positively correlated to ACE activity [85]. Non-O-blood-type individuals have an increased risk of developing SARS-CoV-2 infection and poor outcome and an increase in ACE activity [81]. This may lead to disease progression and abnormal inflammatory and fibrotic processes in COVID-19 [86].

Significant associations between the minor allele *T* of ABO *rs8176746* and *rs495828* and low ACE activity were reported by Chung et al., 2010 [64]. However, we did not find a direct significant association between genotypes of *rs8176746* and *rs495828* with COVID-19 severity. Therefore, we cannot rule out the association of both *rs8176746* and *rs495828*, at least in part, with COVID-19 severity. It could be considered that ABO phenotype and ACE activity are good intermediate phenotypes for mediating the effect of *rs8176746* and *rs495828*. However, our results indicate that the ABO phenotype can predict the severity of COVID-19 than polymorphisms. Our study demonstrates that the AB blood group may be associated with lower odds of severe disease in unadjusted analysis. Our studied SNPs were found to be indirectly related to COVID-19 severity.

Many genome-wide significant associations between genetic variants at the ABO gene locus and SARS-CoV-2 infection and/or COVID-19 disease severity have been reported [63]. *rs657152* shows a perfect correlation with COVID-19 severity among Europeans [54]. *rs912805253* shows correlation with SARS-CoV-2 infection [59]/COVID-19 severity [55] among Europeans. Similarly, *rs505922* shows a correlation with SARS-CoV-2 infection/COVID-19 severity [58,62]. *rs9411378* [56] and *rs879055593* [61] show a correlation with SARS-CoV-2 infection. *rs8176719* [57] and *rs687289* [60] show a correlation with COVID-19 severity.

## 5. Conclusions

In this single-center cohort of Egyptian patients with confirmed COVID-19, the AB blood group showed an unadjusted association with a lower frequency of severe COVID-19, but this association was not independent after adjustment for age, gender, and comorbidities. Male gender and comorbidities predict COVID-19 severity. Rh status and *rs8176746*/*rs495828* were not significantly associated with COVID-19 severity.

## 6. Limitations

Our study limitations include small sample size, cross-sectional design, single-center design, a relatively small number of individuals with the AB phenotype, and a substantial imbalance between the number of severe and non-severe cases. The recruitment strategy for mild and severe cases may introduce selection bias related to age, comorbidity burden, referral pathway, timing, access to care, testing indication, and calendar period. The association between SARS-CoV-2 variants and ABO/Rh blood groups was not examined. Despite these limits, we have addressed an important issue and provided new insights into the impact of ABO/Rh blood groups on SARS-CoV-2 outcomes within the Egyptian population.

## 7. Recommendations

Large-scale multicenter studies with standardized severity definitions, adequate power for rare ABO/genotype categories, adjusted genetic models, and population stratification assessment are needed. More SNPs should be studied to better understand the relation between ABO blood types and COVID-19. Currently, there are no targeted COVID-19 interventions based on patients’ blood groups. Understanding the molecular pathogenesis underlying the associations between ABO blood group and COVID-19 can be translated to COVID-19 prevention or management strategies. Genotyping of other informative ABO variants is needed to predict disease severity. Both genetic and phenotypic markers should be considered in future prediction of COVID-19 severity. Integrating ABO blood typing with clinical and laboratory data may improve risk prediction for COVID-19 patients.

## Figures and Tables

**Table 1:** Characteristics of the severity of COVID-19 cases.

	Non-Severe COVID-19 Cases	Severe COVID-19 Cases
**Oxygen Levels (SpO_2_)**	≥94% on room air	<94% on room air
**Breathing**	Normal breathing rate; no severe difficulty	Marked tachypnea (>30 breaths/min) or shortness of breath
**Lung Involvement**	No abnormal chest imaging	Extensive lung infiltrates (>50% of lung volume) on CT scans
**Systemic Symptoms**	Fever, cough, fatigue, loss of taste/smell, muscle aches	Acute respiratory distress syndrome (ARDS), septic shock, multiorgan failure
**Medical Care**	Can be managed at home or in ambulatory care settings	Requires hospitalization, Intensive Care Unit (ICU) monitoring, or mechanical ventilation

**Table 2:** Demographic and clinical data of the study participants (Total *n* = 306).

Variables		Frequencies	
		Number	Percent (%)
**Age** Mean ± SD (43.98 ± 24.48) Median 44 (0.2–88)	Pediatric	61	19.9
	Adult	245	80.1
**Gender**	Males	117	38.2
	Females	189	61.8
**COVID-19 Severity**	Non-severe	86	28.1
	Severe	220	71.9
**ABO grouping**	A	99	32.4
	B	73	23.9
	O	97	31.7
	AB	37	12.1
**RH grouping**	Negative	31	10.1
	Positive	275	89.9
**Comorbidities**	Hypertension	132	43.1
	DM	85	27.8
	Chronic lung diseases	11	3.6
	Asthma	7	2.3
	CKD	6	2
	Recurrent infections	1	0.3
	Malignancy	10	3.3
	Autoimmune/collagen diseases	1	0.3
	Immunosuppression	4	1.3
	All comorbidities	153	50
**Clinical condition**	COVID-19 contact	184	60.1
	Hospitalization	216	70.6
	ICU Admission	134	43.8
	Oxygen therapy	172	56.2
	Mechanical ventilation	92	30.1
	Respiratory distress	186	60.8
	Pneumonia	203	66.3
	ARDS	163	53.3
	Shock	6	2
	Sepsis	100	32.7
	MOF	52	17
	AKI	40	13.1
	Hepatitis	66	21.6
	Stroke	11	3.6
	Seizures	10	3.3
**CORAD scoring system**	0	39	12.7
	1	5	1.6
	2	10	3.3
	3	18	5.9
	4	63	20.6
	5	126	41.2
	Missing	45	14.7

Continuous data are expressed as mean ± SD and median (range). Categorical data expressed frequencies; n (%). DM: Diabetes Mellitus; CKD: Chronic Kidney Disease; ICU: Intensive Care Unit; ARDS: Acute Respiratory Distress; MOF: Multiorgan Failure; AKI: Acute Kidney Injury; CORAD: COVID-19 Risk Assessment Diagnosis.

**Table 3:** Analysis of participant laboratory findings according to COVID-19 severity.

	Non-Severe COVID-19 (*n* = 86)	Severe COVID-19 (*n* = 220)	Test of Significance	*p*-Values
**HB (g/dL)**	12.45 ± 1.37	10.33 ± 2.09	t = 9.035	<0.001 *
**WBCs (10^3^/UI)**	9.5 (3–18)	14.35 (1.3–87)	z = −7.868	<0.001 *
**Neutrophils (10^3^/uL)**	6.25 (1.4–12)	9.9 (0–40.28)	z = −7.662	<0.001 *
**Lymphocytes (10^3^/uL)**	2.45 (0.4–6.7)	0.9 (0–31)	z = −9.168	<0.001 *
**Platelets (10^3^/uL)**	240 (169–832)	166.4 (13–741)	z = −6.598	<0.001 *
**SGPT (U/L)**	43 (6–136)	42 (4–4202)	t = −0.204	0.838
**SGOT (U/L)**	32 (20–91)	40 (12–6407)	z = −5.218	<0.001 *
**CRP (mg/L)**	3 (1–5)	115 (1–393)	z = −10.775	<0.001 *
**d-dimer (mg/L)**	0.17	10.2 (0–13)	z = −1.637	0.102
**Creatinine (mg/dL)**	0.6 (0.3–1.3)	0.9 (0.3–2.7)	z = −4.993	<0.001 *
**Ferritin (ng/mL)**	Not done	518 (0–2000)	-	-

Continuous data expressed as mean ± SD and median (range). HB: Hemoglobin; WBCs: White Blood Cells; SGPT: serum glutamate pyruvate transaminase; SGOT: serum glutamic-oxaloacetic transaminase; CRP: C-Reactive Protein. Ferritin was tested for sever cases only according to our hospital’s policy. t: Independent samples *t*-test. z: Mann–Whitney U-test. *: Statistically significant (*p* < 0.05).

**Table 4:** Analysis of participant blood grouping according to COVID-19 severity.

	Non-Severe COVID-19 (*n* = 86)	Severe COVID-19 (*n* = 220)	Test of Significance	*p*-Values	OR (LL–UL 95% C.I.)
**Blood group A vs. Non-A**	28 (32.6%)	71 (32.3%)	χ^2^ = 0.022	0.962	0.987 (0.580–1.681)
**Blood group B vs. Non-B**	17 (19.8%)	56 (25.5%)	χ^2^ = 1.101	0.294	1.386 (0.752–2.554)
**Blood group O vs. Non-O**	25 (29.1%)	72 (32.7%)	χ^2^ = 0.382	0.537	1.187 (0.689–2.045)
**Blood group AB vs. Non-AB**	16 (18.6%)	21 (9.5%)	χ^2^ = 4.774	0.029 *	0.462 (0.228–0.934)
**Rh** **negative**	8 (9.3%)	23 (10.5%)	χ^2^ = 0.090	0.764	1.000
**Rh** **positive**	78 (90.7%)	197 (89.5%)			0.878 (0.377–2.047)

C.I.: Confidence interval. OR: Odds ratio. χ^2^: Chi-Square test. *: Statistically significant (*p* < 0.05).

**Table 5:** ABO genotypes among the participants (total *n* = 306). Data shown are frequencies; *n* (%).

Genotype		Frequencies	
		Number	Percent (%)
***rs8176746***	*GG*	204	66.7
	*GT*	92	30.1
	*TT*	10	3.3
***rs495828***	*GG*	205	67.0
	*GT*	97	31.7
	*TT*	4	1.3

**Table 6:** Genotypic distribution of *rs8176746* and *rs495828* among the participants (total *n* = 306) according to ABO blood groups. Data shown are frequencies; *n* (%).

Genotype		A (*n* = 99)	B (*n* = 73)	O (*n* = 97)	AB (*n* = 37)	Test of Significance	*p*-Values
***rs8176746***	*GG*	95 (96.0%)	12 (16.4%)	93 (95.9%)	4 (10.8%)	MC = 210.292	<0.001 *
	*GT*	4 (4%)	52 (71.2%)	3 (3.1%)	33 (89.2%)	MC = 185.806	<0.001 *
	*TT*	0 (0%)	9 (12.3%)	1 (1%)	0 (0%)	MC = 25.089	<0.001 *
***rs495828***	*GG*	37 (37.4%)	70 (95.9%)	87 (89.7%)	11 (29.7%)	χ^2^ = 112.681	<0.001 *
	*GT*	58 (58.6%)	3 (4.1%)	10 (10.3%)	26 (70.3%)	MC = 104.642	<0.001 *
	*TT*	4 (4%)	0 (0%)	0 (0%)	0 (0%)	MC = 8.474	0.037 *

MC: Monte Carlo test. χ^2^: Chi-Square test. *: Statistically significant (*p* < 0.05).

**Table 7:** Genotypic distribution of *rs8176746* and *rs495828* among the participants (total *n* = 306) according to Rh blood groups. Data shown are frequencies; *n* (%).

Genotype		Rh Negative (*n* = 31)	Rh Positive (*n* = 275)	Test of Significance	*p*-Values
***rs8176746***	*GG*	23 (74.2%)	181 (65.8%)	χ^2^ = 0.879	0.348
	*GT*	6 (19.4%)	86 (31.3%)	χ^2^ = 1.882	0.170
	*TT*	2 (6.5%)	8 (2.9%)	FET = 1.106	0.293
***rs495828***	*GG*	24 (77.4%)	181 (65.8%)	χ^2^ = 1.696	0.193
	*GT*	7 (22.6%)	90 (32.7%)	χ^2^ = 1.352	0.250
	*TT*	0 (0%)	4 (1.5%)	FET = 0.457	0.499

FET: Fisher’s exact test. χ^2^: Chi-Square test. *p*-values ≤ 0.05 were judged significant.

**Table 8:** Genotypic and allelic distribution of *rs8176746* and *rs495828* among the participants (total *n* = 306) according to COVID-19 severity. Data shown are frequencies; *n* (%).

Genotypes and Alleles			Non Severe COVID-19 (*n* = 86)		Severe COVID-19 (*n* = 220)		*p*-Values	OR	95% C.I.
			N	%	N	%			
***rs8176746***	**Genotypes**	***GG***	55	64%	149	67.7%	-	1	-
		***GT***	27	31.4%	65	29.5%	0.671	0.889	0.515–1.533
		***TT***	4	4.7%	6	2.7%	0.374	0.554	0.151–2.037
	**^HWE^** ***p***		0.770		0.731		-	-	-
	**Dominant**	***GG***	55	64%	149	67.7%	-	1	-
		***GT*** **+ *TT***	31	36%	71	32.3%	0.529	0.845	0.501–1.426
	**Recessive**	***GG*** **+ *GT***	82	95.3%	214	97.3%	-	1	-
		***TT***	4	4.7%	6	2.7%	0.400	0.575	0.158–2.089
	**Alleles**	***G***	137	79.7%	363	82.5%	-	1	-
		***T***	35	20.3%	77	17.5%	0.413	0.830	0.532–1.296
***rs495828***	**Genotypes**	***GG***	61	70.9%	144	65.5%	-	1	-
		***GT***	25	29.1%	72	32.7%	0.474	1.220	0.708–2.103
		***TT***	0	0%	4	1.8%	FET = 0.580	–	–
	**^HWE^** ***p***		0.115		0.138		-	-	-
	**Dominant**	***GG***	61	70.9%	144	65.5%	-	1	-
		***GT*** **+ *TT***	25	29.1%	76	34.5%	0.360	1.288	0.749–2.214
	**Recessive**	***GG*** **+ *GT***	86	100%	216	98.2%	-	1	-
		***TT***	0	0%	4	1.8%	FET = 0.580	–	–
	**Alleles**	***G***	147	85.4%	360	81.8%	-	1	-
		***T***	25	14.6%	80	18.2%	0.283	1.307	0.802–2.129

C.I.: Confidence interval. HWE: Hardy–Weinberg equation. OR: Odds ratio. FET: Fisher’s exact test. *p*-values ≤ 0.05 were judged significant.

**Table 9:** Univariate and multivariate regression analysis for prediction of severe COVID-19.

Predictors	Univariate Regression				Multivariate Regression			
	*p*-Values	OR	95% C.I.		*p*-Values	OR	95% C.I.	
			Lower	Upper			Lower	Upper
**Age**	0.002 *	1.017	1.006	1.028	0.584	1.004	0.991	1.016
**Female gender**	0.021 *	0.529	0.306	0.909	0.017 *	0.495	0.278	0.883
**Blood group** A	0.962	1.013	0.595	1.725	-	-	-	-
**Blood group** B	0.295	1.386	0.0752	2.554	-	-	-	-
**Blood group** O	0.537	0.842	0.0489	1.451	-	-	-	-
**Blood group** AB	0.032 *	0.768	0.228	0.983	0.112	0.541	0.253	1.154
**Rh positivity**	0.764	1.138	0.488	2.653	-	-	-	-
**Presence of comorbidities**	<0.001 *	5.045	2.857	8.908	<0.001 *	4.688	2.475	8.880

C.I.: Confidence interval. OR: Odds ratio. *: Statistically significant (*p* < 0.05). In univariate analysis, older age, male gender, and the presence of comorbidities were identified as predictors of disease severity, while the AB blood group was protective in this unadjusted model. In multivariate analysis, only male gender and the presence of comorbidities remained as predictors of disease severity.

**Table 10:** Adjusted models for each SNP for age, gender, and comorbidities.

	Univariate Regression				Multivariate Regression			
	*p*-Values	Crude OR	95% C.I.		*p*-Values	Adjust OR	95% C.I.	
			Lower	Upper			Lower	Upper
***rs8176746***								
***GG***		1.000				1.000		
***GT*** **+ *TT***	0.529	0.845	0.501	1.426	0.528	0.834	0.475	1.464
***rs495828***								
***GG***		1.000				1.000		
***GT*** **+ *TT***	0.360	1.288	0.749	2.214	0.448	1.254	0.699	2.251

C.I.: Confidence interval. OR: Odds ratio. *p*-values < 0.05 were judged significant.

## Data Availability

All data are available in the manuscript.

## Abbreviations

The following abbreviations are used in this manuscript: **ACE2**; Angiotensin-Converting Enzyme 2, **AKI**; Acute Kidney Injury, **ARDS**; Acute Respiratory Distress Syndrome, **BA**; Bronchial Asthma, **CRP**: C-reactive protein, **CI**; Confidence Interval, **CKD**; Chronic Kidney Disease, **CLD**; Chronic Liver Disease, **CORAD**; COVID-19 Risk Assessment Diagnosis, **DM**; Diabetes Mellitus, **GWAS**; Genome-Wide Association Studies, **ICU**; Intensive Care Unit, **IQR**; Interquartile Range, **MOF**; Multiorgan Failure, **OR**; Odds Ratio, **SARS-CoV-2**; Severe Acute Respiratory Syndrome Coronavirus-2, **SGOT**; Serum Glutamate Oxaloacetic Transaminase, **SGPT**; Serum Glutamate Pyruvic Transaminase, **S protein**; Spike protein, **SPSS**; Statistical Package for Social Science.

## Appendix A

**Table A1:** Overview of previous studies reporting the role of ABO/Rh blood groups in SARS-CoV-2 susceptibility and/or COVID-19 severity.

Country	Publication Year	Sample Size (Positive SARS-CoV-2 RT-PCR Test)	Type of Study	Main Outcome of Interest	Reported Main Results	Reference No.
**China**	2020	105	Case–control	SARS-CoV-2 susceptibility	Blood group A showed greater susceptibility. No significant associations were detected with other blood groups.	[9]
**Columbia**	2020	95	Cross-sectional	COVID-19 severity	COVID-19 patients with blood groups A or AB are more likely to require mechanical ventilation, renal dialysis, and a longer ICU stay.	[10]
**Denmark**	2020	7422	Cohort	SARS-CoV-2 susceptibility	Blood group O is notably linked to a decreased susceptibility. In young people, healthcare personnel, and those without cardiovascular conditions, blood group O clearly provided relative protection against SARS-CoV-2 susceptibility.	[11]
**Iraq**	2020	1014	Case–control	SARS-CoV-2 susceptibility	Blood group A showed greater susceptibility.	[12]
**Lebanon**	2020	146	Case–control	SARS-CoV-2 susceptibility	Blood group A showed greater susceptibility. Blood group O is linked to a decreased susceptibility.	[13]
**Turkey**	2020	186	Case–control	SARS-CoV-2 susceptibility and COVID-19 severity	Blood groups play a role in increasing susceptibility. Blood group O may provide protection. After infection, blood group type appears to have no impact on COVID-19 clinical outcomes.	[14]
**United Kingdom**	2020	968	Cohort	SARS-CoV-2 susceptibility	Bood group O is more protective than A. ACE inhibitors (ACEIs) may increase susceptibility.	[15]
**Austria**	2021	336	Cohort	SARS-CoV-2 susceptibility	Blood group O is protective, while AB increases the risk. There is a noted association between Lewis Antigens and SARS-CoV-2.	[16]
**Bangladesh**	2021	771	Cross-sectional	COVID-19 severity	Blood group A is linked to higher COVID-19 severity, complications, need for mechanical ventilation and more oxygen, and death. Blood group O has the lowest risk of problems.	[17]
**Brazil**	2021	31	Observational case series and case–control	COVID-19 severity	Blood group A is associated with sever COVID-19 recurrence.	[18]
**Canada**	2021	225556	Cohort	SARS-CoV-2 susceptibility and COVID-19 severity	The O-negative blood type is protective. Individuals with O-negative blood may not exhibit any symptoms.	[19]
**Egypt**	2021	547	Cross-sectional	SARS-CoV-2 susceptibility and COVID-19 severity	Blood group O is regarded as protective. Blood group A is associated with severe forms of pneumonia.	[20]
**India**	2021	2586	Retrospective	SARS-CoV-2 susceptibility	People who are Rh-positive and belong to blood type A are more vulnerable to contracting SARS-CoV-2. The risk of infection is reduced for blood groups O and B.	[21]
**Nigeria**	2021	297	Cross-sectional	SARS-CoV-2 susceptibility	AB and B blood groups showed greater susceptibility. Blood group O is protective.	[22]
**Saudi Arabia**	2021	404	Cohort	SARS-CoV-2 susceptibility	Blood group O provides protection.	[23]
**Spain**	2021	483	Cohort	SARS-CoV-2 susceptibility and COVID-19 severity	Blood group O provides protection. Blood group B patients experience more post-COVID-19 complications.	[24]
**Sudan**	2021	557	Case–control	SARS-CoV-2 susceptibility and COVID-19 severity	Blood group A showed greater susceptibility. Blood group O is protective. Rh-positive status has a favorable influence on blood type O but a detrimental effect on A.	[25]
**Turkey**	2021	39,850	Retrospective	COVID-19 severity	Blood group A COVID-19 patients showed increased rates of intensive care unit (ICU) admissions. Rh status did not affect hospital and ICU admission.	[26]
**Turkey**	2021	220	Cohort	SARS-CoV-2 susceptibility	Younger people and those with blood group O are more. Curfews and travel restrictions may not have applied to this age group.	[27]
**USA**	2021	4968	Cohort	COVID-19 severity	Admission rates differ among patients based on their ABO blood groups, while discharge rates are consistent across groups. Compared to blood type O, blood type A is associated with death.	[28]
**Saudi Arabia**	2022	373	Cohort	SARS-CoV-2 susceptibility and COVID-19 severity	Blood group B showed greater susceptibility, with mild to moderate COVID-19 presentation. Blood group O is protective.	[29]
**Spain**	2022	1399	Retrospective	COVID-19 severity	The O blood group showed a significantly reduced risk of hospitalization and ICU admission.	[30]
**Japan**	2022	461	Case–control	SARS-CoV-2 susceptibility	Blood group O is protective than others.	[31]
**United Arab** **Emirates (UAE)**	2022	303	Retrospective	COVID-19 severity	Patients with blood group B had a decreased age-related risk of pneumonia and mortality than those with O. Patients with blood group AB had significantly higher creatinine levels than others.	[32]
**Iraq**	2023	671	Cross-sectional	SARS-CoV-2 susceptibility	Blood group A is linked to susceptibility. Blood group O is protective. Rh-negative blood is protective.	[33]
**USA**	2023	596	Retrospective	COVID-19 severity	ABO blood type is not linked to the severity or mortality of COVID-19. Rhesus factor status is associated with the severity and mortality. Rh-negative patients have an increased risk of mortality.	[34]
**Iran**	2024	101	Cohort	SARS-CoV-2 susceptibility and COVID-19 severity	Blood group A+ is linked to susceptibility, severity and mortality. Blood group O+ might be protective.	[35]
**Pakistan**	2024	120	Cross-sectional	COVID-19 severity	Blood group A is significantly linked to the severity.	[36]
**Saudi Arabia**	2024	2302	Cross-sectional	SARS-CoV-2 susceptibility and COVID-19 severity	ABO and Rh blood types do not predict susceptibility or mortality.	[37]
**USA**	2024	9416	Cohort	COVID-19 severity	ABO blood type was not linked to severity across the Delta, Alpha, and Omicron variants.	[38]
**Bangladesh**	2025	100	Case–control	SARS-CoV-2 susceptibility and COVID-19 severity	Higher levels of natural anti-A and anti-B antibodies may provide protection. Variations in blood groups suggest potential innate immune factors that could influence the disease severity.	[39]
**Canada**	2025	128	Retrospective	COVID-19 severity	Blood types A and AB predict more severity. The observation of higher adipsin levels in patients with blood types A and AB compared to those with O and B calls for further investigation in larger prospective studies.	[40]
**China**	2025	823	Retrospective	COVID-19 severity	A greater risk of severe or serious COVID-19 development is linked to non-B blood types.	[41]
**Ethiopia**	2025	570	Retrospective	COVID-19 severity	Severity or mortality is unrelated to variations in ABO blood types.	[42]
**Ghana**	2025	77	Case–control	SARS-CoV-2 susceptibility and COVID-19 severity	Blood group O is protective. Blood group A is linked to more risk.	[43]
**India**	2025	185	Retrospective	SARS-CoV-2 susceptibility and COVID-19 severity	Blood group A is linked to more risk. Patients with blood group AB experienced the most severe clinical presentation.	[44]
**India**	2025	120	Retrospective	SARS-CoV-2 susceptibility and COVID-19 severity	Blood groups A and AB are linked to more susceptibility and greater severity. Susceptibility is slightly higher in Rh-positive patients.	[45]
**Iran**	2025	271	Cross-sectional	COVID-19 severity	ABO and Rh blood types do not predict COVID-19 severity, ICU admission, or mechanical ventilation need.	[46]
**Pakistan**	2025	700	Case–control	SARS-CoV-2 susceptibility and COVID-19 severity	Blood group A is a risk factor. Blood group O offered protection. A strong relationship was noted between comorbidities (such as hypertension and diabetes) and severe outcomes in COVID-19 patients.	[47]
**Saudi Arabia**	2025	446	Retrospective cohort	COVID-19 severity	The thrombotic risk linked to SARS-CoV-2 may be influenced by ABO blood group phenotypes, particularly group A. Combining ABO typing with RNAemia screening may enhance risk assessment and provide targeted thromboprophylaxis for patients with COVID-19, even though no clear connection was found between blood group and spike mutations or RNAemia.	[48]

The table emphasizes that population-specific differences exist that affect the impact of ABO/Rh blood types on SARS-CoV-2 susceptibility, COVID-19 severity, and the underlying pathogenesis.
